# Role of CASP7 polymorphisms in noise-induced hearing loss risk in Han Chinese population

**DOI:** 10.1038/s41598-021-81391-5

**Published:** 2021-01-19

**Authors:** Yanmei Ruan, Jinwei Zhang, Shiqi Mai, Wenfeng Zeng, Lili Huang, Chunrong Gu, Keping Liu, Yuying Ma, Zhi Wang

**Affiliations:** 1 Key Laboratory of Occupational Environment and Health, Guangzhou Twelfth People’s Hospital, 1 Tianqiang St., Huangpu West Ave., Guangzhou, 510620 Guangdong China; 2 The Institute of Occupational and Environmental Health, Guangzhou Twelfth People’s Hospital Affiliated to Guangzhou Medical University, Guangzhou, 510620 China; 3 Department of Occupational Health Monitoring, Guangzhou Twelfth People’s Hospital, Guangzhou, 510620 China; 4 Department of Anesthesia, People’s Liberation Army Southern Theater Air Force Hospital, Guangzhou, 510000 China

**Keywords:** Genotype, Mutation, Sequencing, Health occupations, Risk factors

## Abstract

Genetic factors and gene-environment interaction may play an important role in the development of noise induced hearing loss (NIHL). 191 cases and 191 controls were selected by case–control study. Among them, case groups were screened from workers exposed to noise in binaural high-frequency hearing thresholds greater than 25 dB (A). Workers with hearing thresholds ≤ 25 dB (A) in any binaural frequency band were selected to the control group, based on matching factors such as age, exposure time to noise, and operating position. The blood samples from two groups of workers were subjected to DNA extraction and SNP sequencing of CASP3 and CASP7 genes using the polymerase chain reaction ligase detection reaction method. Conditional logistic regression correction was used to analyze the genetic variation associated with susceptibility to NIHL. There was an association between rs2227310 and rs4353229 of the CASP7 gene and the risk of NIHL. Compared with the GG genotype, the CC genotype of rs2227310 reduced the risk of NIHL. Compared with CC genotype, the TT genotype of rs4353229 reduced the risk of NIHL. Workers carrying the rs2227310GG and rs4353229CC genotype had an increased risk of NIHL compared to workers without any high-risk genotype. There were additive interaction and multiplication interaction between CASP7rs2227310 and CNE, and the same interaction between CASP7rs4353229 and CNE. The interaction between the CASP7 gene and CNE significantly increased the risk of NIHL. The genetic polymorphisms of CASP7rs2227310GG and CASP7rs4353229CC were associated with an increased risk of NIHL in Han Chinese population and have the potential to act as biomarkers for noise-exposed workers.

## Introduction

Noise refers to sound that can cause physical and mental discomfort to human beings. It exists widely in the manufacturing environment and can cause health damage to workers and reduce production efficiency. With the progress of China's industries, productive noise has become one of the main occupational exposure in the working environment. Occupational noise deafness is the leading occupational disease in China, and occupational noise is a serious health hazard to the occupational population. Noise can cause specific hearing damage to human body and non-specific damage to other organ systems. Specific damage is mainly noise-induced hearing loss (NIHL). Non-specific damage includes impairs on the nervous system, cardiovascular system, endocrine system and other physical functions^1,2^.

Noise-induced hearing loss is related to many factors, including noise exposure levels^3^, noise exposure time^3^, noise properties^4^, interaction with other factors (high temperature, organic solvents, etc.)^5,6^, individual health-related behaviors (personal protective measures, smoking, drinking etc.)^7^, individual sensitivity^8^, individual health status (hypertension, hyperlipemia, etc.)^9^ and so on. Genetic factors and gene–environment interactions may also play important roles in the development of NIHL.

Researchers have found in long-term animal experiments and population studies that even under the same noise exposure, the degree of hearing threshold displacement varies among different experimental animals and populations. This shows that individuals with noise-induced hearing loss (NIHL) have different susceptibility^8^.

However, some researchers have reported that cochlear hair cell damage and death can cause NIHL in individuals^10^. Apoptosis is part of the methods that cause cochlear hair cell death, and cochlear hair cell death is attributed to cell-independent and orderly death controlled by specific genes^11,12^. Studies have shown that cysteine aspartic protease (Caspase) is a cysteine protease with specific aspartic acid, of which the activation is the main step leading to apoptosis. Under the influence of apoptosis signals, caspase is activated by gradual hydrolysis, and the cleavage of cell structure and functional proteins can give rise to apoptosis^13^. Caspase3 and Caspase7 have highly similar functions and substrate specificity. They are the most important effectors in the process of apoptosis, and also the converging point of many apoptosis stimulation signals. Their activation marks irreversible apoptosis^14^. It has also been shown that the genetic variation of CASP3 gene is related to the risk of NIHL, and the joint effect of working time and CASP3 polymorphism may affect the risk of NIHL^15^. In this study, we assume that the Caspase7 genes may be associated with the risk of noise-induced hearing loss in the Chinese population. We selected 191 NIHL and 191 noise-exposed workers as the research subjects, and performed genetic analysis of 14 single nucleotide polymorphisms (SNP) in their CASP3 and CASP7 genes, and analyzed their interaction with environmental behavioral factors.

## Results

### Participant data equilibrium analysis

There were 191 cases and 191 controls in this study, with a mean age of 32.19 ± 6.41 years and 31.88 ± 5.92 years, respectively. There were no significant differences in age, noise exposure time, noise exposure intensity, cumulative noise exposure [CNE, dB (A)·Year], and body mass index (BMI) between the case and the control group (P > 0.05) (The matching of case group and control group is shown in the Additional file 1: Table S2).

### Analysis of basic characteristics of participants

The differences in basic characteristics of the study subjects between the two groups are shown in Table 1. Their demographic characteristics include Age, Educational level, Marital status, Personal monthly income, Noise exposure time, Wear noise protection products, Smoking frequency, Drinking frequency, Wear headphones to listen to music/watch videos, Call time per day, Length of one's sleep, BMI, Total cholesterol, Triglyceride, and CNE, which were not significantly different (P > 0.05). There were significant differences between the two groups on different diet taste (P < 0.05). Compared with the control group, more workers in the case group had a salty taste.

**Table 1:** Distribution of different characteristics in the two groups.

Individual characteristics	Cases (n = 191)	Controls (n = 191)	χ^2^	*P*-value
	n (%)	n (%)		
**Age (year)**				
≤ 25	27 (14.1)	32 (16.8)	0.571	0.752
25–35	106 (55.5)	105 (55.0)		
≥ 35	58 (30.4)	54 (28.3)		
**Marital status**				
Unmarried	57 (29.8)	64 (33.5)	0.593	0.441
Married	134 (70.2)	127 (66.5)		
**Personal monthly income (RMB)**				
≤ 3000	7 (3.7)	9 (4.7)	0.525	0.769
3001–8000	119 (62.3)	113 (59.2)		
≥ 8000	65 (34.0)	69 (36.1)		
**Noise exposure time (year)**				
1–5	45 (23.8)	44 (23.0)	0.042	0.979
6–10	48 (25.1)	47 (24.6)		
> 10	98 (51.3)	100 (52.4)		
**Wear noise protection products**				
Occasionally wear	127 (66.5)	115 (60.2)	1.624	0.203
Standard wear	64 (33.5)	76 (39.8)		
**Smoking frequency (cigarette/day)**				
0~	97 (50.8)	113 (59.2)	2.954	0.228
1~	66 (34.6)	52 (27.2)		
11~	28 (14.7)	26 (13.6)		
**Drinking frequency (times/week)**				
0~	120 (62.8)	131 (68.6)	3.391	0.183
1~	52 (27.2)	37 (19.4)		
2~	19 (9.9)	23 (12.0)		
**Diet taste**				
Light	69 (36.1)	89 (46.6)	28.422	< 0.001
Salty	90 (47.1)	53 (27.7)		
Partial sweet	9 (4.7)	11 (5.8)		
Partial oil	21 (11.0)	17 (8.9)		
Other	2 (1.0)	21 (11.0)		
**Wear headphones to listen to music/watch videos**				
Never listen	91 (47.6)	78 (40.8)	2.647	0.266
Sometimes listen	90 (47.1)	97 (50.8)		
Often listen	10 (5.2)	16 (8.4)		
**Call time per day (min)**				
0	22 (11.5)	21 (11.0)	1.142	0.767
0~	126 (66.0)	129 (67.5)		
15~	27 (14.1)	30 (15.7)		
30~	16 (8.4)	11 (5.8)		
**Length of one's sleep (h)**				
< 7.0	70 (36.6)	71 (37.2)	1.310	0.519
7~	116 (60.7)	118 (61.8)		
9~	5 (2.5)	2 (1.0)		
**BMI (kg/m**^**2**^**)**				
< 18.5	7 (3.7)	7 (3.7)	4.079	0.130
18.5~	117 (61.3)	135 (70.7)		
24.0~	67 (35.1)	49 (25.7)		
**Total cholesterol**				
Normal	163 (85.3)	166 (86.9)	0.197	0.657
Abnormal	28 (14.7)	25 (13.1)		
**Triglyceride**				
Normal	171 (89.5)	174 (91.1)	0.269	0.604
Abnormal	20 (10.5)	17 (8.9)		
**CNE [dB(A)·year]**				
< 90	58 (30.9)	59 (30.9)	0.334	0.954
90~	88 (46.1)	90 (47.1)		
95~	42 (22.0)	39 (20.4)		
100~	2 (1.0)	3 (1.6)		

### Correlation analysis between SNP and NIHL risk

**Figure 1:**
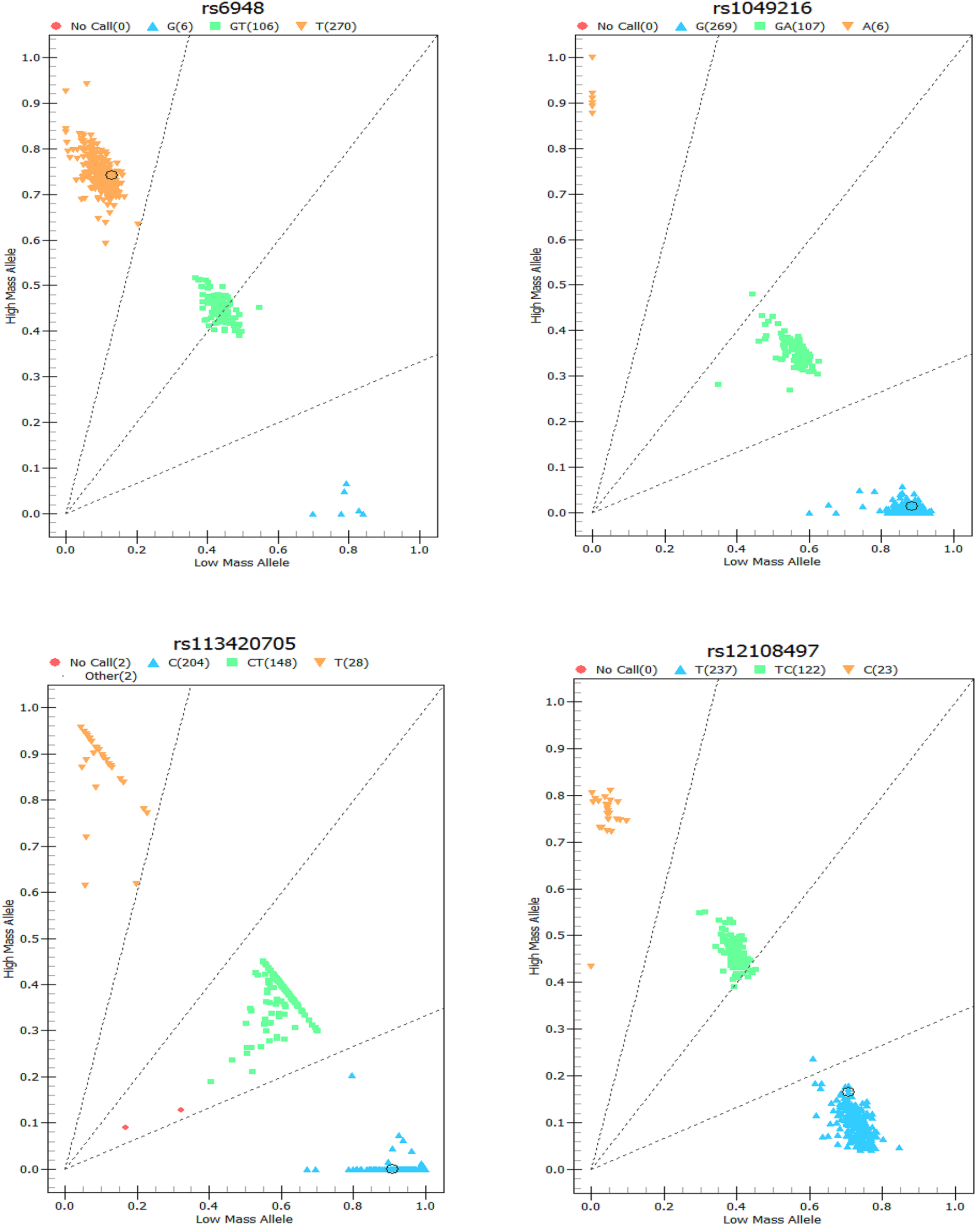

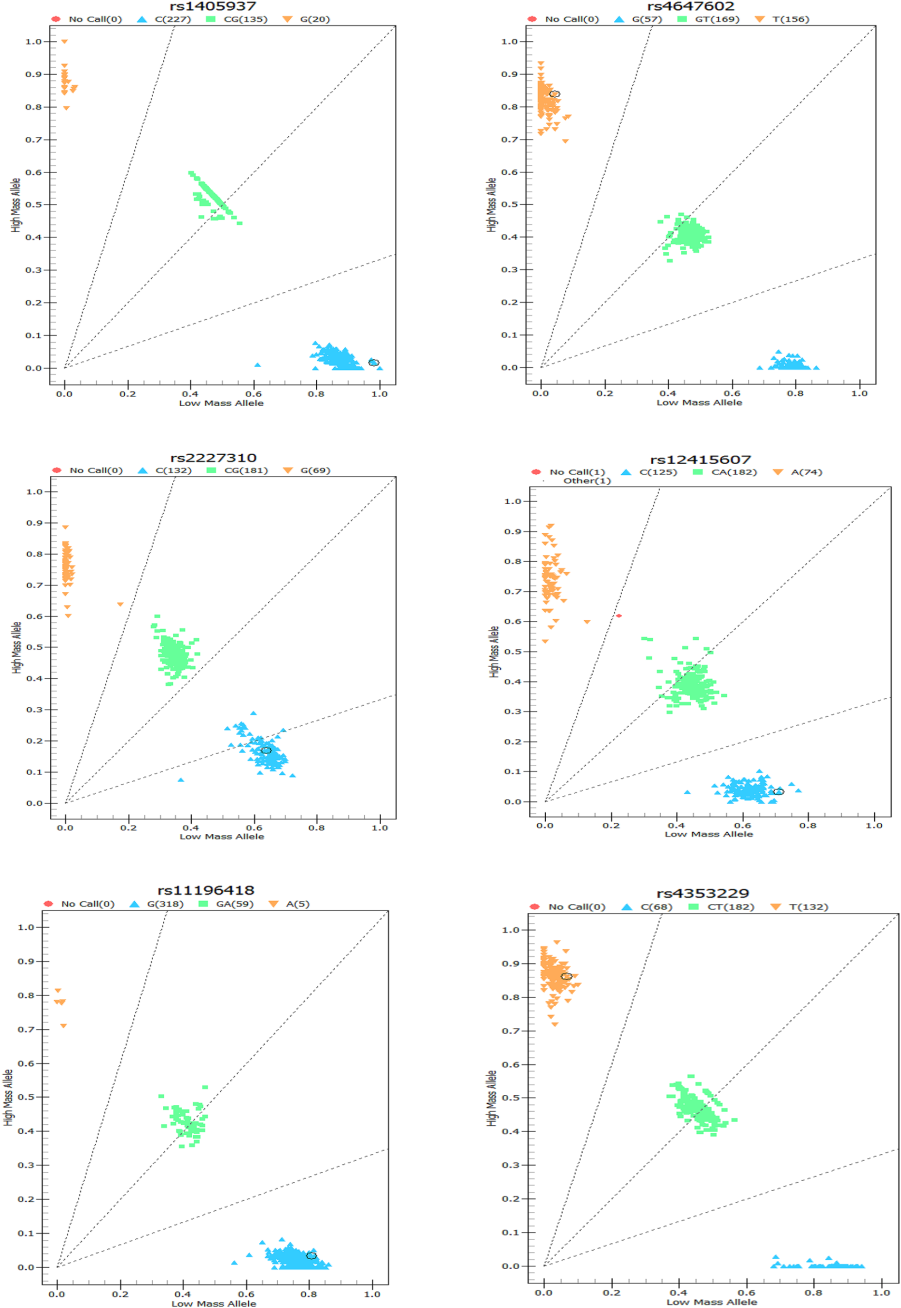

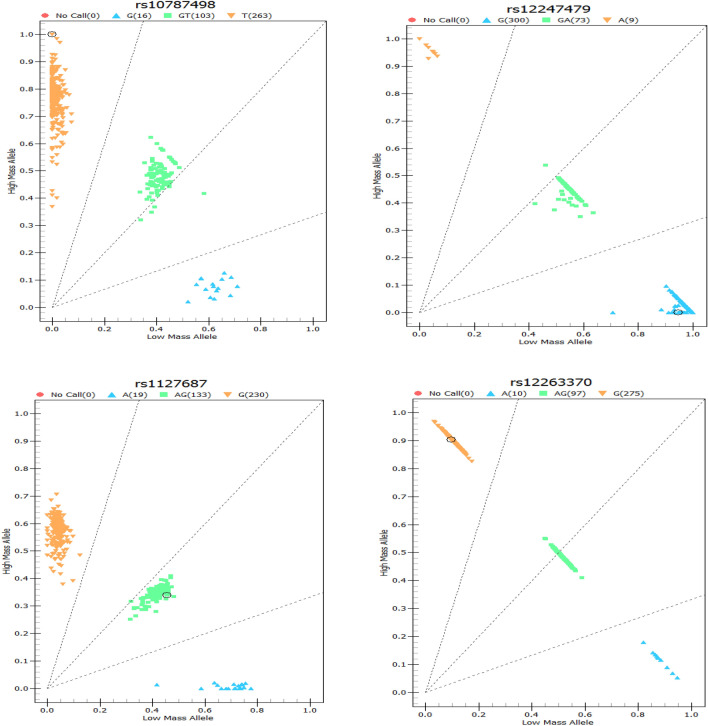
Gene coordinates for SNP sites.

The gene coordinates map of each SNP is shown in figure file: Fig. 1. We performed logistic regression analysis on 14 genotypes of the CAPS3 and CAPS7 genes (see Additional file 1: Table S3), and found that there was an association between rs2227310 and rs4353229 of the CASP7 gene and the risk of NIHL (Table 2). Compared with the GG genotype, the risk of NIHL in the rs2227310 CC genotype was reduced (OR = 0.480, 95% CI 0.262–0.880). Similarly, it was also found that in the recessive genetic model, compared with the GG genotype, the risk of NIHL carrying the (CC + CG) genotype was reduced (OR = 0.545, 95% CI 0.314–0.946). The risk of NIHL in rs4353229 TT genotype was reduced (OR = 0.490, 95% CI 0.267–0.899) comparing with CC genotype. It was also found in the recessive genetic model that comparing with the CC genotype, the risk of NIHL in the rs4353229 (TT + CT) genotype was reduced (OR = 0.560, 95% CI 0.322–0.974). We classified the rs2227310GG and rs4353229CC genotype as high-risk genotype and then calculated the number of high-risk genotypes in the combined genotype. We found that workers carrying the rs2227310GG and rs4353229CC genotype had an increased risk of NIHL in comparison with workers who did not carry any high-risk genes (OR = 1.795, 95% CI 1.031–3.123). The association of rs2227310 and rs4353229 with NIHL remained significant after Benjamini–Hochberg correction (P < 0.05).

**Table 2:** Association between SNP and risk of NIHL.

SNP	Genetic model	Genotype	Cases (n = 191)	Controls (n = 191)	P^a^	OR (95% CI)^a^
rs2227310	Additive	GG	42 (22.0)	27 (14.1)		1.000
		CC	62 (32.5)	70 (36.6)	0.018	0.480 (0.262–0.880)
		CG	87 (45.5)	94 (49.2)	0.039	0.548 (0.310–0.969)
	Dominant	GG + CG				1.000
		CC	129 (67.5)	121 (63.4)	0.605	0.890 (0.572–1.384)
	Recessive	GG				1.000
		CC + CG	149 (78.0)	164 (85.9)	0.031	0.545 (0.314–0.946)
rs4353229	Additive	CC	41 (21.5)	27 (14.1)		1.000
		TT	62 (32.5)	70 (36.6)	0.021	0.490 (0.267–0.899)
		CT	88 (46.1)	94 (49.2)	0.050	0.566 (0.320–1.001)
	Dominant	CC + CT				1.000
		TT	129 (67.5)	121 (63.4)	0.305	0.890 (0.572–1.384)
	Recessive	CC				1.000
		TT + CT	150 (78.5)	164 (85.9)	0.040	0.560 (0.322–0.974)
Risk genotype^b^		0	149 (78.0)	164 (85.9)		1.00
		1	1 (0.5)	0 (0.0)	–	–
		2	41 (21.5)	27 (14.1)	0.039	1.795 (1.031–3.123)

^a^Adjusted for age, marital status, personal monthly income, noise exposure time, wear noise protection products, smoking frequency, drinking frequency, diet taste, wear headphones to listen to music/watch videos, call time per day, length of one's sleep, BMI, total cholesterol, triglyceride and CNE.

^b^rs2227310 GG and rs4353229 CC genotype were classified as high-risk genotype; the number represents the numbers of the two genotypes within the combined genotype.

### Linkage disequilibrium and haplotype analysis of CASP7 gene

We explored the degree of linkage unevenness of these two SNP sites, which is usually expressed by the linkage disequilibrium coefficient D′ and the correlation coefficient r^2^. The results showed that D′ = 1, r^2^ = 0.995 (see Additional file 1: Table S4), rs2227310 and rs4353229 have a strong linkage disequilibrium. The haplotype analysis showed that there were no statistically significant differences between the CASP7 gene haplotype and NIHL (P > 0.05) (see Additional file 1: Table S5).

### The potential interaction between SNP and environmental factors

The results of the interaction analysis between the two sites of CNE and CASP7 were shown in Table 3. There are additive interaction and multiplication interaction between CASP7rs2227310 and CNE, AP is 0.695 (0.323–1.067), Pmul = 0.011; Similarly, CASP7rs4353229 and CNE also have additive interaction and multiplication interaction, AP is 0.675(0.280–1.070), Pmul = 0.015.

**Table 3:** The risk analysis of the occurrence of NIHL by the interaction between GASP7 gene SNP and cumulative noise exposure.

Variable	SNP	Genotype	Cases, n (%)	Controls, n (%)	OR^a^ (95% CI)	*P*_mul_^a^	AP^a^ (95% CI)	RERI^a^ (95% CI)
**CNE [dB(A)·year]**	rs2227310							
≤ 92		CC + CG	77 (40.3)	82 (42.9)	1.000			
> 92		CC + CG	20 (10.5)	17 (8.9)	0.987 (0.531–1.838)	0.011	0.695 (0.323–1.067)	2.557 (-0.997–6.111)
≤ 92		GG	72 (37.7)	82 (42.9)	1.134 (0.520–2.474)			
> 92		GG	22 (11.5)	10 (5.2)	3.679 (1.338–10.117)			
**CNE [dB(A)·year]**	rs4353229							
≤ 92		TT + CT	77 (40.3)	82 (42.9)	1.000			
> 92		TT + CT	20 (10.5)	17 (8.9)	1.003 (0.539–1.865)	0.015	0.675·(0.280–1.070)	2.368·(− 1.037 to 5.774)
≤ 92		CC	73 (38.2)	82 (42.9)	1.135·(0.520–2.474)			
> 92		CC	21 (11.0)	10 (5.2)	3.505·(1.269–9.687)			

*P*_*mul*_ it was calculated using the product interaction term in Logistic regression analysis.

^a^Adjusted for age, marital status, personal monthly income, noise exposure time, wear noise protection products, smoking frequency, drinking frequency, diet taste, wear headphones to listen to music/watch videos, call time per day, length of one's sleep, BMI, total cholesterol, triglyceride.

The interaction between the CASP7 gene and CNE significantly increases the risk of NIHL. Compared with workers who do not carry dangerous genotypes and are exposed to CNE ≤ 92 [dB(A)·Year], workers who carry dangerous genotypes and CNE > 92 [dB(A)·Year] have a significantly increased risk of NIHL. Compared with workers carrying the CASP7rs2227310CC + CG genotype of CNE ≤ 92 [dB(A) Year], workers carrying GG and CNE > 92 [dB(A)·Year] increased the risk of NIHL (OR = 3.679, 95% CI 1.338–10.117, P < 0.05). Secondly, Compared with workers carrying the CASP7rs4353229TT + TC genotype of CNE ≤ 92 [dB(A)·Year], workers carrying CC and CNE > 92 [dB(A)·Year] increased the risk of NIHL(OR = 3.505, 95% CI 1.269–9.687, P < 0.05) (Table 3).

### The potential interaction between SNP and SNP

We used Multifactor dimensionality reduction (MDR) method to explore the potential interactions within genes, but no statistical significances were found (see Additional file 1: Table S6).

## Discussion

Noise-induced hearing loss (NIHL) has been ubiquitous all over the world. In China, workers in the automotive industries usually work at least eight hours per day and rest only one day per week. The high intensity of noise generated during die casting, stamping and welding can greatly increase the risk of hearing damage within workers. Previous studies had found that smoking and drinking were associated with the risk of noise-induced hearing loss^16,17^. Wang and Yang found that polymorphism of catalase gene is related to NIHL susceptibility, identified CAT is a NIHL susceptibility gene when noise exposure levels are taken into account^18,19^. Some studies have found that SNPs in the HSP70, EYA4, CDH23, GRHL2, and DFNA5 genes are associated with genetic susceptibility to NIHL in human^20–22^.

We matched noise-exposed workers in the same working position according to age, noise exposure age, noise exposure intensity, BMI and CNE in the NIHL group and the control group. Both groups of workers are exposed to similar environments and are comparable. The differences in hearing loss mainly reflect differences in genetic susceptibility. By genotyping and analyzing 14 SNP in CASP3 and CASP7 genes, we found that rs2227310 and rs4353229 of CASP7 genes were associated with the risk of NIHL.

Caspase 7 was an important regulatory factor and executive factor in the process of apoptosis, and it played an important role in the development of tumors. Lee SY and other studies found that CASP7 rs2227310 polymorphic variant alleles increased the risk of lung cancer in recessive and dominant models^23^. Yan has also studied that rs2227310, rs3124740, and rs12415607 of CASP7 may be an increased risk of cancer^24^. Wang MY and other studies discovered that CASP7 rs4353229TT genotype may be associated with reduced risk of gastric cancer^25^. Studies have reported that CASP7 gene mutations in the Chinese population may modulate overall survival and progression-free survival rate of patients with advanced non-small cell lung cancer platinum chemotherapy^26^. There are few studies exploring the association between CASP7 gene polymorphisms and noise-induced hearing loss, and the majority of studies are related to tumors. In our study, we referred to previous studies and analyzed genotypes according to Additive model, dominant model, and recessive model^15^. We found that genetic variation of rs2227310 was associated with increased risk of NIHL, while subjects with their variant alleles (CC or CG) reduced the risk of NIHL. Similarly, we found that genetic variation of rs4353229 was associated with increased risk of NIHL, while subjects with their alleles of variation (TT or CT) were associated with reduced risk of NIHL and increased genetic risk of NIHL. The rs2227310GG and rs4353229CC genotypes were defined as high-risk genotypes. By calculating the number of high-risk genotypes, we found that the risk of NIHL in people with two risk genotypes was 1.795 times higher than those without risk genotypes. These findings suggest that genetic variation in the CASP7 gene may alter the risk of NIHL.

Previous studies have shown that NIHL is a disease caused by a combination of genes and environmental factors, with noise as the main environmental factor. The relationship between genes and NIHL susceptibility was affected by noise exposure^20^. We calculated cumulative noise exposure (CNE) according to current international noise exposure standards (ISO-1999, 2013). It was assumed that the effect of noise exposure on hearing is proportional to the duration of exposure multiplied by the intensity of energy exposed. Therefore, we also conducted a hierarchical analysis through CNE. According to the results of previous studies and the analysis in Table 3, we analyzed the interaction between CNE and genes with a statistically significant recessive model^27^. There were additive interaction and multiplication interaction between CASP7 rs2227310 and CNE; similarly, the same interaction is also found between CASP7 rs4353229 and CNE. The interaction between the CASP7 gene and CNE significantly increased the risk of workers suffering from NIHL. Compared with workers who did not carry dangerous genotypes and were exposed to CNE ≤ 92 [dB(A)·Year], workers carried dangerous genotypes and CNE > 92 [dB(A)·Year] had a significantly increased risk of NIHL. This phenomenon may be due to the fact that people exposed to noise are more susceptible to NIHL under strong noise levels^28^. These findings suggest that gene-environment interaction may play an important role in the risk of NIHL.

There was a linkage disequilibrium (LD) between SNPs. Our study found that rs2227310 and rs4353229 had a strong linkage disequilibrium (D′1.00, r20.995). CASP7 rs2227310 is a missense mutation site in the exon region. The mutation of C to G resulted in its encoded amino acid aspartic acid mutated into glutamic acid, which may lead to abnormal structure and function of CASP7α subtype, thereby accelerated the process of apoptosis^29^. Rs4353229 of CASP7 is a miRNA binding site located in the 3′UTR region. MicroRNA (miRNA) is a small class of non-coding RNA molecules. MiRNA regulate gene expression by binding to the 3′untranslated region (UTR) of their target mRNA, leading to mRNA cleavage or translation inhibition^13^. Sequence variations (such as SNPs) located in the 3′-UTR of miRNA target genes may also eliminate or weaken microRNA targets or produce imperfect sequences that match microRNA seed. This disrupts the microRNA-mRNA interaction and affects the expression of microRNA targets and the expression of CASP7^30^. However, there may be some limitations in our research. First of all, the research subjects we selected were all male Han workers (fewer women in the automobile manufacturing industries), and there may be gender and ethnic differences. Secondly, the sample size in the case groups and control groups was small, and we may have missed some significant outcomes that could only occur with large sample size. Thirdly, our selection of subjects may lead to selection bias.

In summary, our study found that the rs2227310 CC and rs4353229 TT genotype of the CAPS7 gene may be less susceptible to the development of NIHL. Genetic variations of CASP7 and their interactions with cumulative noise exposure were associated with genetic susceptibility to NIHL and may modify the risk of noise induced hearing loss.

## Methods

### Participants

In 2019, we selected noise-exposed workers who had undergone occupational health checks from a number of automobile manufacturers in Guangzhou as participants, and the study was conducted from March to October in China. The selected research subjects have relatively fixed job positions and are less mobile in the production process. The study included 191 NIHL workers and 191 hearing-normal workers, and no workers were exposed to other occupational hazards. We selected 191 cases of noise exposed workers with binaural high-frequency hearing thresholds greater than 25 dB (A). The control group was matched according to the following criteria: (1) same enterprises, types of work and operating positions as the case group; (2) binaural arbitrary frequency bands (including 500, 1000, 2000, 3000, 4000, 6000 Hz) hearing thresholds less than or equal to 25 dB (A); (3) same age (± 3 years), same noise exposure time (± 1 year).

The inclusion criteria for the subjects were as follows.

1. Cumulative time of occupational noise exposure [noise exposure time ≥ 8 h/day or 40 h/week, noise intensity ≥ 80 dB (A)] > 1 year;
2. male and Han;
3. age: 18–45 years old.

The exclusion criteria were as follows:

1. Exposure to explosives or head injuries within 1 month prior to physical examination;
2. family history of hearing loss;
3. otitis or other otological diseases;
4. fever or common infections (flu, diarrhea and hepatitis, etc.);
5. history of taking ototoxic drugs;
6. participants with bone conduction audiometry suggestive of conductive deafness.

The physical examination was performed by occupational health examiners in accordance with standard protocol for each participant. Height, weight, and pure tone audiometry were measured. We also inquired about the contact situation of other occupational hazards. We used EDTA anticoagulant negative pressure glass tubes to collect peripheral whole blood of empty-stomach subjects. The biochemical indexes such as triglyceride and total cholesterol were measured by Beckman AU-680 automatic biochemical analyzer. The blood collection tube has a test tube number that can be one-to-one corresponded to the physical examination number, which ensures the consistency of the blood sample, the physical examination result and the questionnaire. Blood samples were temporarily stored and safely transported in a mobile refrigerator after collected.

Questionnaire items included general conditions, occupational history, personal history, past history. The general situation included age, gender, ethnicity, marital status and personal monthly income status. Occupational history included noise exposure time and wearing conditions of noise protection equipment. Personal history included smoking frequency, drinking frequency, length of one's sleep and diet taste, wear headphones to listen to music/watch videos, call time per day. Past history including history of head trauma, exposure to explosive operations, ear disease, long-term use of ototoxic drugs, and history of infectious diseases, was primarily used to rule out other factors that may affect hearing function. The survey was conducted by professionally trained investigators. They collected information by conducting face-to-face surveys and inquiries with each subject using a questionnaire.

### Variable definition

During the questionnaire survey, the investigator explained to the research objects that the contents need to be filled out one by one. Smoking refers to smoking an average of at least one cigarette per day and continuing to smoke for more than 6 months. Alcohol consumption is defined as drinking at least once a week on average for more than 1 year. Definition of dietary taste: light taste means that foods with higher salt content, sugar content, fat, and spicy content are not preferred, compared to people around them; salty means that salt is often added or additional salt needs to be added when dining; partial sweetness means that people around you have a higher preference for beverages and desserts with higher sugar content; partial oil means higher preference for foods with higher energy content than people around you or the need for meals with extra cooking oil. The standard wearing of noise protection equipment refers to workers wearing noise protection equipment strictly when entering the working environment according to the instructions and operational requirements; occasionally wearing refers to not wearing noise protection equipment as required. According to the frequency of noise workers wearing headphones listening to music/watching videos after getting off from work, they are divided into three types: not listening, sometimes listening and often listening. Among them, noise workers who almost don't wear headphones to listen to music/watch videos after getting off from work is defined as not listening; wearing headphones everyday to listen to music/watch videos is defined as regular listening, and the rest are classified as sometimes listening. Total cholesterol ≥ 6.2 mmol/L is defined as abnormal TC. Triglyceride ≥ 2.3 mmol/L is defined as abnormal TG. We also use Epidata3.1 software to double-enter the questionnaire data. The whole process is to ensure data integrity and validity.

On the basis of the “Diagnosis of Occupational Noise Deafness” (GBZ 49-2014) and relevant regulations and standards, specialized occupational health doctors performed at least three pure-tone hearing tests (the pure-tone hearing threshold test is performed in accordance with GB/T7583 and GB/T16403). Hearing thresholds of both ears were determined in increments of 5 dB in 500 dB, 1000 Hz, 2000 Hz, 3000 Hz, 4000 Hz, and 6000 Hz frequencies. According to GB/T7582–2004, the results were modified by age and gender. The PTA defines the hearing threshold at high frequencies as the average of each ear at 3000, 4000, and 6000 Hz. The hearing threshold at speech frequencies is defined as the average of 500, 1000, and 2000 Hz per ear. All subjects were required to avoid noise exposure for more than 48 h before conducting audiometry.

The study conducted noise detection in accordance with “Measurement of physical factors in the workplace-Part 8: Noise” (GBZ/T189.8-2007). We measured noise after elaborative observation and detailed investigation on spatial distribution, processing procedure, and equipment layout of the working environment in these factories. We used EDGE individual noise dosimeter produced by the British company CASELLA to evaluate noise intensity. In this study, Noise exposure was evaluated with A-weighted energy equivalent continuous sound pressure level (Lex.8h) according to the National Criteria of Measurement of Noise in the Workplace (GBZ/T189.8-2007) (China, 2007). Cumulative noise exposure (CNE) was calculated as CNE = Lex.8h + 10 log T (Formula 2), where T means years of noise exposure.

### SNP selection and genotyping

1. **Screening of candidate gene SNP**
  - Find the gene name in NCBI-SNP (http://www.ncbi.nlm.nih.gov/snp/). Opening Gene View, refreshes after clicking Clinical Source and gene region. Select functional SNP sites of Promoter proxy (upstream variant 2 KB), 5′UTR, Exon (missense, synonymous), 3′UTR region (Relevant optimization parameters are MAF in CHB > 0.05, based on HapMap or 1000 Genomes database) in this gene.
  - Mark disease-susceptible results by referring to relevant literature
  - Predict the function of the screened SNP (http://snpinfo.niehs.nih.gov/).
  - Perform LD analysis of the above SNP and mark the full linkage site of R2 = 1.
    (http://asia.ensembl.org/Homo_sapiens/Tools/LD?db=core).
2. **The SNP inclusion criteria are as following:**
  1. Functional SNP sites located in Promoter proxy (upstream variant 2 KB), 5′UTR, Exon (missense, synonymous), 3′UTR;
  2. MAF in CHB > 0.05;
  3. The linkage disequilibrium value of r2 is > 0.80;
  4. Genetic balance test (Hardy–Weinberg) P value > 0.05. In this study, We selected 14 SNP sites from CASP3 and CASP7 (see Additional file 1: Table S1).
3. **Primer design and dilution**
  Sort rs numbers of the sites before detection, and input the rs numbers to http://agenacx.com/ for primer design. According to the results of the operation, select and determine the appropriate primer design scheme, and order primers. The PCR primer was diluted to 100 μM, and a PCR primer mixture was prepared according to 1:200. Extension primers were diluted according to the dilution table. Prepare the EXT primer mix at 1:25. After the extension primer mixture was prepared, 2 μl was diluted 25-fold for mass spectrometry. Adjust the extension primer ratio of individual sites according to the test results.
4. **DNA extraction**
  Sample DNA was extracted using a ThermoFisher automated magnetic bead extractor. For blood samples, the Magpure Buffy Coat DNA Midi KF Kit was used. The NanoDrop8000 instrument was used for OD value detection and a 1.25% agarose gel electrophoresis. After passing the DNA quality test, the sample DNA was transferred to a 96-well plate and stored at − 20 °C.
5. **Agena MassArray system genotyping steps**
  A target fragment containing the SNP site to be detected was amplified by a PCR reaction. Shrimp alkaline phosphatase (SAP enzyme) was then used to remove the remaining deoxyribonucleoside triphosphate (dNTP) and primers in the PCR system. Then single base extension primers were added, the 3′terminal base of which is close to the SNP site and is completely complementary to the base on the target fragment. Four types of ddNTP were used instead of dNTP. The probe extended only one base at the SNP site, and the ddNTP on the connection corresponds to the allele of the SNP site. Matrix-assisted laser desorption ionization time-of-flight mass spectrometry (MALDI-TOF MS) was used to detect the molecular weight difference between the extended product and the unextended primer, and the base at this point was determined.
  The PCR master mix was configured and oscillated at low speed. We added 4 μl PCR master mixes to each well of the 384-well plate, and mixed them after 1 μl of template DNA (20 ng/μl) was added. The PCR reaction plate was placed on the PCR instrument and then the program was started. After the PCR reaction was completed, the PCR products were treated with SAP to remove free dNTPs from the system. Next, we prepared alkaline phosphatase treatment in a new 1.5 ml EP tubes, following by adding the SAP mix to a 384-well PCR reaction plate. After centrifugation, the SAP reaction program was performed, and then a single base extension reaction was activated after the completion of alkaline phosphatase treatment. Subsequently, we prepared a single base extension reaction solution in a new 1.5 ml EP tube, and added the EXTEND Mix to the 384-well reaction plate. Again with centrifugation, an extension reaction procedure was performed. The cation exchange resin was used to remove Na+, Mg^2+^, K+ and other salt ions after the PCR reaction, so as to avoid excessive salt peaks in the analysis spectrum produced by mass detection, which would affect the result judgment. The PCR product plate was centrifuged for 5 min (4000 r/min). And 19 μl of ultrapure water was added to each reaction well and centrifuged for 1 min. Resin was applied on the top, and the PCR product plate was left to dry at room temperature for 15–30 min. Afterwards, they were mixed up for 40 min or 1 h. The sample was then micro-loaded onto a SpectroCHIP with a Mass Array Nanodispenser to prepare a co-crystallized film of the chip matrix and the sample. The prepared chip was put into a mass spectrometer (MassARRAY Analyzer 4 System) for detection, and Typer 4.0 software was used to obtain the original data and the cluster map, and check the integrity and accuracy of the data file.

### Statistical analysis

Categorical variables were expressed in frequency (%) and analyzed by Pearson's χ^2^ independent test. Quantitative variables obeying normal distribution were expressed as mean ± standard deviation (M ± SD) and analyzed by Student's t test. We performed a χ^2^ goodness-of-fit test on the frequency of each genotype tested to verify that it complies with Hardy–Weinberg Equilibrium (HWE). Conditional logistic regression was used to correct statistically significant confounding factors in the study. The OR (Odds Ratio) value and its 95% CI (Confidence interval) were used to analyze the correlation between the genes of SNP and the risk of NIHL. Multiple comparisons corrections were carried out with Benjamini—Hochberg correction. The environment–gene-based multiplication interaction was calculated using the product interaction term in Logistic regression analysis. If the gene-environment product term P < 0.05, then the two factors have a multiplicative interaction. The additive interaction effect is evaluated by excess relative risk due to interaction (RERI) and the attributable proportion due to interaction (AP). RERI (95% CI) contains 0 means there is no additive interaction between the two factors; RERI (95% CI) does not contain 0 means there is additive interaction. The greater the absolute value of RERI is, the stronger interaction between the two factors. AP (95% CI) contains 0 indicates that there is no additive interaction between the two factors; AP (95% CI) does not contain 0 means there is additive interaction between the two factors. The greater the absolute value of AP is, the stronger interaction between the two factors. Haplotype analysis and linkage disequilibrium analysis were conducted using SHEsis platform^31^. Multifactor dimensionality reduction (MDR) method was used to explore potential interactions between genes. Statistical analysis was performed using SAS version 9.2 (SAS INSTITUTE INC, Cary, NCSU, USA). The MDR method used MDR version 3.0.2 (Computational Genetics Laboratory of the University of Pennsylvania, Philadelphia, PA, USA). We considered all significant statistical tests with p value < 0.05.

### Ethical approval statement

The study was approved by Guangzhou Twelfth People’s Hospital ethics committee. The committee believed that the design and plan of the study fully takes into account the principles of safety and fairness, and its research content did not cause any harm to the participants. The recruitment of participants was based on the principle of voluntary and informed consent. The project team protected the rights and privacy of participants in accordance with relevant national regulations, and there was no conflict of interest between the research content and the research results.

### Written informed consent

This study did not cause any harm or risk to participants. All participants have signed written informed consent.

### Relevant guidelines and regulations

All methods were carried out in accordance with relevant guidelines and regulations (Declaration of Helsinki).

## Supplementary Information

Supplementary Tables.
