# Supplementary material for: Role of CASP7 polymorphisms in noise-induced hearing loss risk in Han Chinese population
Source: Sci Rep. 2021 Jan 19;11:1803. doi: 10.1038/s41598-021-81391-5 (PMC7815823; doi:10.1038/s41598-021-81391-5)
Supplement: Supplementary file 1 — Supplementary Tables. [file 41598_2021_81391_MOESM1_ESM.doc]

**Role of CASP7 polymorphisms in noise-induced hearing loss risk in Han Chinese population**

**Authors**

Yanmei Ruan^2^, Jinwei Zhang^1^, Shiqi Mai^1^, Wenfeng Zeng^3^, Lili Huang^3^, Chunrong Gu^4^, Keping Liu^2^, Yuying Ma^2^, Zhi Wang^1,2^.

1. Department of Occupational Health Management, Guangzhou Twelfth People's Hospital, Guangzhou 510620, China.
2. Department of Occupational Health Management, Guangzhou Twelfth People's Hospital Affiliated to Guangzhou Medical University, Guangzhou 510620, China.

3. Department of Occupational Health Monitoring, Guangzhou Twelfth People's Hospital, Guangzhou 510620, China.

4. Department of anesthesia, People's Liberation Army Southern Theater Air Force Hospital, Guangzhou 510000, China.

**^*^ Corresponding author**

Zhi Wang, PhD, MD, Professor

Department of Occupational Health Management, Guangzhou Twelfth People's Hospital,

1 Tianqiang St., Huangpu West Ave., Guangzhou, Guangdong, 510620 China.

Email: zhi_wang@outlook.com

**Email**

Yanmei Ruan：1076739591@qq.com

Jinwei Zhang：zhang_jinwei@126.com

Shiqi Mai：maisq_crystal@126.com

Wenfeng Zeng：wf_zeng@163.com

Lili Huang：294555453@qq.com

Chunrong Gu：847761477@qq.com

Keping Liu：1096554472@qq.com

Yuying Ma：yyma1218@163.com

Zhi Wang：zhi_wang@outlook.com

**Table S1. Basic information, primer and probe sequences of selected SNP.**

|  | **allele** | **MAF IN CHB** | **Function Prediction** | **Region** | **Primers for PCR** | **Probes for detecting variations** | ***P*_HWE_** |
| --- | --- | --- | --- | --- | --- | --- | --- |
| **CASP3** |  |  |  |  |  |  |  |
| rs6948 | G/T | 0.199 | -- | 3' UTR | F:GGAGGCCTCCCGGGCTGAG | TG:GGAGGCCTCCCGGGCTGAGG  TT:GGAGGCCTCCCGGGCTGAGT | 0.107 |
| rs1049216 | G/A | 0.209 | miRNA binding site | 3' UTR | R:TGAAAAAGTTAAACATTGAAGTAA | TG:TGAAAAAGTTAAACATTGAAGTAAC  TA:TGAAAAAGTTAAACATTGAAGTAAT | 0.107 |
| rs113420705 | C/T | 0.32 | -- | 5' UTR | F:AGCCTCCTCATACCTTC | TC:AGCCTCCTCATACCTTCC  TT:AGCCTCCTCATACCTTCT | 0.400 |
| rs12108497 | T/C | 0.282 | TFBS | promoter | R:GGACTCTGTGACTATAAAAGATG | TT:GGACTCTGTGACTATAAAAGATGA  TC:GGACTCTGTGACTATAAAAGATGG | 0.607 |
| rs1405937 | C/G | 0.277 | TFBS | promoter | F:CCCCCAGGGACCCCATGGCA | TC:CCCCCAGGGACCCCATGGCAC  TG:CCCCCAGGGACCCCATGGCAG | 0.848 |
| rs4647602 | G/T | 0.288 | TFBS | promoter | F:CTGCAGGGCCGAAAA | TG:CTGCAGGGCCGAAAAG  TT:CTGCAGGGCCGAAAAT | 0.914 |
| **CASP7** |  |  |  |  |  |  |  |
| rs2227310 | C/G | 0.427 | nsSNP | Exon-missense | F:AGGGAGCACGGAAAAGA | TC:AGGGAGCACGGAAAAGAC  TG:AGGGAGCACGGAAAAGAG | 0.610 |
| rs12415607 | C/A | 0.417 | TFBS | promoter | R:TTGAGTACATGCTTAGTGGTC | TC:TTGAGTACATGCTTAGTGGTCG  TA:TTGAGTACATGCTTAGTGGTCT | 0.491 |
| rs11196418 | G/A | 0.117 | TFBS | promoter | R:CCCAAACACACAGATTCTAGTT | TG:CCCAAACACACAGATTCTAGTTC  TA:CCCAAACACACAGATTCTAGTTT | 0.598 |
| rs4353229 | C/T | 0.427 | miRNA binding site | 3' UTR | F:ACATGCAACAGAAGTGAC | TC:ACATGCAACAGAAGTGACC  TT:ACATGCAACAGAAGTGACT | 0.611 |
| rs10787498 | G/T | 0.194 | miRNA binding site | 3' UTR | F:CAGTGGTAGAGTCATGT | TG:CAGTGGTAGAGTCATGTG  TT:CAGTGGTAGAGTCATGTT | 0.251 |
| rs12247479 | G/A | 0.112 | miRNA binding site | 3' UTR | R:CCATTGGTGGTCCTAA | TG:CCATTGGTGGTCCTAAC  TA:CCATTGGTGGTCCTAAT | 0.129 |
| rs1127687 | A/G | 0.204 | miRNA binding site | 3' UTR | F:CAGCCATGACAAGAACAAA | TA:CAGCCATGACAAGAACAAAA  TG:CAGCCATGACAAGAACAAAG | 0.881 |
| rs12263370 | A/G | 0.141 | TFBS | promoter | F:GGAAGTAAGCCACCGCGCCT | TA:GGAAGTAAGCCACCGCGCCTA  TG:GGAAGTAAGCCACCGCGCCTG | 0.193 |

Table S2. Comparison of matching factors between case group and control group.

| Matching factors | Cases（n=191） |  | Controls（n=191） | ***t*** | ***P*** |
| --- | --- | --- | --- | --- | --- |
| Age(year) | 32.19±6.41 |  | 31.88±5.92 | 0.484 | 0.629 |
| Noise exposure time (year) | 9.36±5.52 |  | 9.44±5.54 | 0.146 | 0.884 |
| Noise intensity [dB(A)] | 83.08±2.74 |  | 82.91±2.92 | 0.569 | 0.569 |
| CNE [dB(A).year] | 91.64±4.73 |  | 91.53±4.89 | 0.220 | 0.826 |
| BMI (kg/m^2^) | 22.64±2.89 |  | 22.35±2.78 | 0.990 | 0.323 |

Table S3. Association between SNP and risk of NIHL.

| Gene | SNP | Genetic model | Genotype | Cases  (n=191) | Controls(n=191) | *P*^#^ | OR（95%CI）^#^ |
| --- | --- | --- | --- | --- | --- | --- | --- |
| **CASP3** | rs6948 | Additive | TT | 139（72.8） | 131（68.6） |  | 1.000 |
|  |  |  | GG | 4（2.1） | 2（1.0） | 0.483 | 1.875  (0.323~10.863) |
|  |  |  | GT | 48（25.1） | 58（30.4） | 0.296 | 0.778  (0.486~1.246) |
|  |  | Dominant | GG+GT vs TT | 52（27.2） | 60（31.4） | 0.394 | 1.221  (0.771~1.933) |
|  |  | Recessive | GG vs TT+GT | 187（97.9） | 189（99.0） | 0.446 | 0.506  (0.087~2.924) |
|  |  | Hyper dominant | TT+GG vs GT | 143（74.9） | 133（69.6） | 0.276 | 0.770  (0.481~1.232) |
|  | rs1049216 | Additive | GG | 138（72.3） | 131（68.6） |  | 1.000 |
|  |  |  | AA | 4（2.1） | 2（1.0） | 0.529 | 1.758  (0.304~10.164) |
|  |  |  | GA | 49（25.7） | 58（30.4） | 0.348 | 0.799  (0.499~1.278) |
|  |  | Dominant | AA+GA vs GG | 53（27.7） | 60（31.4） | 0.442 | 1.197  (0.757~1.895) |
|  |  | Recessive | AA vs GA+GG | 187（97.9） | 189（99.0） | 0.490 | 0.539  (0.094~3.110) |
|  |  | Hyper dominant | GG+AA vs GA | 142（74.3） | 133（69.6） | 0.326 | 0.791  (0.495~1.263) |
|  | rs113420705 | Additive | CC | 101（52.9） | 103（53.9） |  | 1.000 |
|  |  |  | TT | 18（9.4） | 10（5.2） | 0.146 | 1.871  (0.803~4.357) |
|  |  |  | CT | 72（37.7） | 76（39.8） | 0.941 | 1.017  (0.656~1.577) |
|  |  | Dominant | TT+CT vs CC | 90（47.1） | 86（45.0） | 0.606 | 0.895  (0.589~1.364) |
|  |  | Recessive | TT vs CC+CT | 173（90.6） | 179（93.7） | 0.141 | 0.538  (0.236~1.227) |
|  |  | Hyper dominant | CC+TT vs CT | 119（62.3） | 113（59.2） | 0.784 | 0.942  (0.615~1.443) |
|  | rs12108497 | Additive | CC | 13（6.8） | 10（5.2） |  | 1.000 |
|  |  |  | TT | 118（61.8） | 119（62.3） | 0.582 | 0.779  (0.319~1.900) |
|  |  |  | TC | 60（31.4） | 62（32.5） | 0.649 | 0.807  (0.320~2.036) |
|  |  | Dominant | CC+TC vs TT | 73（38.2） | 72（37.7） | 0.752 | 0.933  (0.607~1.434) |
|  |  | Recessive | CC vs TT+TC | 178（93.2） | 181（94.8） | 0.595 | 0.788  (0.328~1.896) |
|  |  | Hyper dominant | TT+CC vs TC | 131（68.6） | 129（67.5） | 0.956 | 1.013  (0.648~1.582) |
|  | rs1405937 | Additive | CC | 112（58.6） | 115（60.2） |  | 1.000 |
|  |  |  | GG | 11（5.8） | 9（4.7） | 0.605 | 1.286  (0.496~3.334) |
|  |  |  | CG | 68（35.6） | 67（35.1） | 0.692 | 1.093  (0.704~1.697) |
|  |  | Dominant | GG+CG vs CC | 79（41.4） | 76（39.8） | 0.612 | 0.896  (0.587~1.368) |
|  |  | Recessive | GG vs CC+CG | 180（94.2） | 182（95.3） | 0.650 | 0.805  (0.315~2.055) |
|  |  | Hyper dominant | GG+CC vs CG | 123（64.4） | 124（64.9） | 0.758 | 1.071  (0.694~1.650) |
|  | rs4647602 | Additive | TT | 79（41.4） | 77（40.3） |  | 1.000 |
|  |  |  | GG | 31（16.2） | 26（13.6） | 0.486 | 1.251  (0.667~2.345) |
|  |  |  | GT | 81（42.4） | 88（46.1） | 0.693 | 0.913  (0.582~1.433) |
|  |  | Dominant | GG+GT vs TT | 112（58.6） | 114（59.7） | 0.957 | 1.012  (0.663~1.543) |
|  |  | Recessive | GG vs TT+GT | 160（83.8） | 165（86.4） | 0.362 | 0.763  (0.426~1.366) |
|  |  | Hyper dominant | GG+TT vs GT | 110（57.6） | 103（53.9） | 0.478 | 0.860  (0.567~1.305) |
| **CASP7** | rs2227310 | Additive | GG | 42（22.0） | 27（14.1） |  | 1.000 |
|  |  |  | CC | 62（32.5） | 70（36.6） | 0.018 | 0.480  (0.262~0.880) |
|  |  |  | CG | 87（45.5） | 94（49.2） | 0.039 | 0.548(0.310~0.969) |
|  |  | Dominant | GG+CG vs CC | 129（67.5） | 121（63.4） | 0.605 | 0.890  (0.572~1.384) |
|  |  | Recessive | GG vs CC+CG | 149（78.0） | 164（85.9） | 0.031 | 0.545(0.314~0.946) |
|  |  | Hyper dominant | GG+CC vs CG | 104（54.5） | 97（50.8） | 0.226 | 0.769  (0.502~1.177) |
|  | rs12415607 | Additive | CC | 59（30.9） | 66（34.6） |  | 1.000 |
|  |  |  | AA | 38（19.9） | 36（18.8） | 0.653 | 1.146  (0.633~2.074) |
|  |  |  | CA | 94（49.2） | 88（46.1） | 0.625 | 1.126  (0.701~1.808) |
|  |  | Dominant | AA+CA vs CC | 132（69.1） | 124（64.9） | 0.678 | 0.911  (0.588~1.413) |
|  |  | Recessive | AA vs CC+CA | 153（80.1） | 154（80.6） | 0.802 | 0.935  (0.554~1.580) |
|  |  | Hyper dominant | AA+CC vs CA | 97（50.8） | 102（53.4） | 0.669 | 1.095  (0.723~1.658) |
|  | rs11196418 | Additive | GG | 158（82.7） | 160（83.8） |  | 1.000 |
|  |  |  | AA | 3（1.6） | 2（1.0） | 0.388 | 2.298  (0.347~15.225) |
|  |  |  | GA | 30（15.7） | 29（15.2） | 0.464 | 1.245  (0.693~2.237) |
|  |  | Dominant | AA+GA vs GG | 33（17.3） | 31（16.2） | 0.371 | 0.770  (0.435~1.364) |
|  |  | Recessive | AA vs GG+GA | 188（98.4） | 189（99.0） | 0.421 | 0.462  (0.070~3.035) |
|  |  | Hyper dominant | AA+GG vs GA | 161（84.3） | 162（84.8） | 0.509 | 1.217  (0.679~2.181) |
|  | rs4353229 | Additive | CC | 41（21.5） | 27（14.1） |  | 1.000 |
|  |  |  | TT | 62（32.5） | 70（36.6） | 0.021 | 0.490  (0.267~0.899) |
|  |  |  | CT | 88（46.1） | 94（49.2） | 0.050 | 0.566  (0.320~1.001) |
|  |  | Dominant | CC+CT vs TT | 129（67.5） | 121（63.4） | 0.605 | 0.890  (0.572~1.384) |
|  |  | Recessive | CC vs TT+CT | 150（78.5） | 164（85.9） | 0.040 | 0.560  (0.322~0.974) |
|  |  | Hyper dominant | CC+TT vs CT | 103（53.9） | 97（50.8） | 0.265 | 0.785  (0.513~1.202) |
|  | rs10787498 | Additive | GG | 7（3.7） | 9（4.7） |  | 1.000 |
|  |  |  | TT | 134（70.2） | 129（67.5） | 0.513 | 1.421  (0.495~4.079) |
|  |  |  | GT | 50（26.2） | 53（27.7） | 0.773 | 1.176  (0.390~3.543) |
|  |  | Dominant | GG+GT vs TT | 57（29.8） | 62（32.5） | 0.360 | 1.236  (0.785~1.946) |
|  |  | Recessive | GG vs TT+GT | 184（96.3） | 182（95.3） | 0.574 | 1.350  (0.474~3.844) |
|  |  | Hyper dominant | GG+TT vs GT | 141（73.8） | 138（72.3） | 0.484 | 0.844  (0.525~1.357) |
|  | rs12247479 | Additive | GG | 155（81.2） | 145（75.9） |  | 1.000 |
|  |  |  | AA | 3（1.6） | 6（3.1） | 0.288 | 0.459  (0.109~1.929) |
|  |  |  | GA | 33（17.3） | 40（20.9） | 0.228 | 0.720  (0.422~1.229) |
|  |  | Dominant | AA+GA vs GG | 36（18.8） | 46（24.1） | 0.147 | 1.459  (0.875~2.432) |
|  |  | Recessive | AA vs GA+GG | 188（98.4） | 185（96.9） | 0.327 | 2.049  (0.489~8.582) |
|  |  | Hyper dominant | AA+GG vs GA | 158（82.7） | 151（79.1） | 0.260 | 0.736  (0.432~1.254) |
|  | rs1127687 | Additive | GG | 118（61.8） | 112（58.6） |  | 1.000 |
|  |  |  | AA | 9（4.7） | 10（5.2） | 0.779 | 1.151  (0.431~3.079) |
|  |  |  | AG | 64（33.5） | 69（36.1） | 0.576 | 0.881  (0.566~1.372) |
|  |  | Dominant | AA+AG vs GG | 73（38.2） | 79（41.4） | 0.664 | 1.099  (0.718~1.680) |
|  |  | Recessive | AA vs GG+AG | 182（95.3） | 181（94.8） | 0.705 | 0.829  (0.314~2.188) |
|  |  | Hyper dominant | AA+GG vs AG | 127（66.5） | 122（63.9） | 0.539 | 0.872  (0.564~1.349) |
|  | rs12263370 | Additive | GG | 132（69.1） | 143（74.9） |  | 1.000 |
|  |  |  | AA | 4（2.1） | 6（3.1） | 0.620 | 0.717  (0.192~2.677) |
|  |  |  | AG | 55（28.8） | 42（22.0） | 0.216 | 1.356  (0.837~2.195) |
|  |  | Dominant | AA+AG vs GG | 59（30.9） | 48（25.1） | 0.305 | 0.785  (0.494~1.247) |
|  |  | Recessive | AA vs GG+AG | 187（97.9） | 185（96.9） | 0.544 | 1.502  (0.404~5.583) |
|  |  | Hyper dominant | AA+GG vs AG | 136（71.2） | 149（78.0） | 0.198 | 1.371  (0.848~2.216) |

^#^adjusted for age, marital status, personal monthly income, noise exposure time, wear noise protection products, smoking frequency, drinking frequency, diet taste, wear headphones to listen to music / watch videos, call time per day, length of one's sleep, BMI, total cholesterol, triglyceride and CNE.

Table S4. Analysis of linkage disequilibrium of CASP 7 gene SNP

| SNP | rs2227310 | rs4353229 |
| --- | --- | --- |
| rs2227310 |  | D’ 1.000 |
| rs4353229 | R^2^ 0.995 |  |

Table S5. Haplotypes of CASP 7 gene and risk analysis of NIHL

| Haplotypes | Case | Control | *P* | OR (95% CI) |
| --- | --- | --- | --- | --- |
|  | n(%) | n(%) |  |  |
| CT | 211(55.2) | 234(61.3) |  | 1.000 |
| GC | 170(44.5) | 148(38.7) | 0.100 | 1.274(0.955~1.700) |
| GT | 1(0.3) | 0(0.0) | - | - |

Table S6. The best combination models identified by MDR.

| No. | Best model* | Training balanced  accuracy (%) | Testing balanced  accuracy (%) | *P* | Cross-validation  consistency |
| --- | --- | --- | --- | --- | --- |
| 1 | rs2227310 | 0.5416 | 0.4921 | 0.9055 | 5/10 |
| 2 | rs1405937, rs10787498 | 0.5859 | 0.4764 | 0.7688 | 5/10 |
| 3 | rs4647602, rs12415607, rs10787498 | 0.6041 | 0.4817 | 0.8206 | 5/10 |
